# Classification of Non-Functional Requirements From IoT Oriented Healthcare Requirement Document

**DOI:** 10.3389/fpubh.2022.860536

**Published:** 2022-03-18

**Authors:** Iqra Khurshid, Salma Imtiaz, Wadii Boulila, Zahid Khan, Almas Abbasi, Abdul Rehman Javed, Zunera Jalil

**Affiliations:** ^1^Department of Software Engineering, International Islamic University, Islamabad, Pakistan; ^2^Robotics and Internet-of-Things Laboratory, Prince Sultan University, Riyadh, Saudi Arabia; ^3^Department of Cyber Security, Air University, Islamabad, Pakistan

**Keywords:** non-functional requirements, healthcare, classification, machine learning, requirement document

## Abstract

Internet of Things (IoT) involves a set of devices that aids in achieving a smart environment. Healthcare systems, which are IoT-oriented, provide monitoring services of patients' data and help take immediate steps in an emergency. Currently, machine learning-based techniques are adopted to ensure security and other non-functional requirements in smart health care systems. However, no attention is given to classifying the non-functional requirements from requirement documents. The manual process of classifying the non-functional requirements from documents is erroneous and laborious. Missing non-functional requirements in the Requirement Engineering (RE) phase results in IoT oriented healthcare system with compromised security and performance. In this research, an experiment is performed where non-functional requirements are classified from the IoT-oriented healthcare system's requirement document. The machine learning algorithms considered for classification are Logistic Regression (LR), Support Vector Machine (SVM), Multinomial Naive Bayes (MNB), K-Nearest Neighbors (KNN), ensemble, Random Forest (RF), and hybrid KNN rule-based machine learning (ML) algorithms. The results show that our novel hybrid KNN rule-based machine learning algorithm outperforms others by showing an average classification accuracy of 75.9% in classifying non-functional requirements from IoT-oriented healthcare requirement documents. This research is not only novel in its concept of using a machine learning approach for classification of non-functional requirements from IoT-oriented healthcare system requirement documents, but it also proposes a novel hybrid KNN-rule based machine learning algorithm for classification with better accuracy. A new dataset is also created for classification purposes, comprising requirements related to IoT-oriented healthcare systems. However, since this dataset is small and consists of only 104 requirements, this might affect the generalizability of the results of this research.

## 1. Introduction

One of the most important tasks of developing high-quality software is gathering the right requirements and ensuring no missing requirements. Often during the process of Requirement Engineering (RE), more attention is given to eliciting the functional requirements than non-functional requirements. This results in the poor quality end product and results in loss of cost, effort, and even failure of the project. Non-functional requirements explain the important quality attributes (1, 2) and constraints (3) that must be implemented in the system. If these requirements are left ignored, the architecture is not designed properly (4). Thus, classifying the non-functional requirements becomes a mandatory task in the RE process for designing the architecture design and performing other related activities accordingly (4, 5). Traditionally, non-functional requirements are identified and extracted manually which is an erroneous process (5, 6) and laborious (7). The non-functional requirements are intertwined with the functional requirements in RE documents, not properly structured. Therefore, proper identification and categorization are required (8). Since the architect needs to know the types of non-functional requirements, non-functional requirements must be classified properly (9). Currently, the trend of using ML algorithms for classifying the non-functional requirements is gaining attention (8). However, there is still a need to classify the non-functional requirements accurately. By using automated techniques, the chance of missing the non-functional requirements is reduced, classification accuracy can be improved, and time and effort are also saved. IoT possesses a vital role in the health care systems of the present era. It includes a set of devices, which help store, process, and transfer data to achieve smart services (10–13). Healthcare systems, which are IoT-oriented, provide monitoring services of patients' data and help take immediate steps in an emergency (14–16). Thus, it is critical to ensure that these smart healthcare systems are extremely secure and perform reliably so that patients' sensitive data is not only kept protected from manipulation and attacks but also transferred entirely and safely in a timely fashion (17–19). In order to ensure that the IoT-oriented healthcare system possesses all these important non-functional requirements, it is necessary to extract all the non-functional requirements from the requirement document of the IoT-oriented healthcare system in the RE phase. Machine learning-based techniques are adopted to implement non-functional requirements in smart health care systems. However, attention needs to be given to the first classification of the non-functional requirements from the requirement document to ensure that non-functional requirements are not missed in the RE phase. By performing this classification task automatically, the chances of missing non-functional requirements will be reduced, and the probability of developing a high-performing and secure IoT-oriented healthcare system will increase (20).

In this research, the main focus is on finding machine learning algorithm and relevant features which helps in the classification of non-functional requirements with higher accuracy. For features extraction, Bag of Words (BOW) and Term Frequency-Inverse Document Frequency (TF-IDF) are adopted. The algorithms considered by this study are Logistic Regression (LR), Support Vector Machine (SVM), Multinomial Naïve Bayes (MNB), K-Nearest Neighbors (KNN), ensemble, Random Forest (RF), and hybrid KNN rule-based ML algorithms. The two main distinct additions here are the ensemble and hybrid classifiers.

The existing studies have limitations. Some studies have reported low performance (1, 6, 21). The types of non-functional requirements considered by some studies are very less (4, 22, 23). The dataset adopted by different studies has a limited number of non-functional requirements (2, 9, 21). Some studies adopted the manual process of validation which may have chances of error (21).

This article makes the following contributions.

- Creation of dataset, which includes requirements related to IoT-oriented healthcare system. The requirements included in this dataset belong to 8 categories: Accuracy, Reliability, Security and Privacy, Performance, Compatibility, Usability, Functional, and Maintainability. The numbers of requirements in this dataset are 104. IoT-oriented healthcare system requirement documents create this dataset (24, 25). Since there are no currently such dataset available that contains labeled requirements related to IoT-oriented healthcare systems, this will serve as an aid for future research purposes in automating the classification of non-functional requirements from IoT-oriented healthcare system requirement documents.
- Development of a novel hybrid KNN rule-based machine learning algorithm, which provides better classification accuracy than traditional machine learning algorithms like SVM, KNN, RF, MNB, LR, and ensemble. This novel hybrid machine learning algorithm helps classify non-functional requirements from IoT-oriented healthcare system requirement documents with an average classification accuracy of 75.9%.
- Provision of features relevant to non-functional requirements that the IoT-oriented healthcare system must possess. These features will help the researchers to create better classifiers by including them in machine learning-based techniques.

This research article is structured in the following manner. Section 2 describes the relevant work conducted earlier in the literature related to the classification of the non-functional requirements. Section 3 describes the research methodology which is adopted in this research. Section 4 shows the experiment portion. Section 5 presents the results and discussion. Section 6 provides the conclusion and highlights possible future directions.

## 2. Literature Review

In literature, many studies have highlighted the problem of ignored and missed non-functional requirements in the RE process (8, 21). Non-functional requirements are sometimes hidden and not clearly stated, due to which it becomes difficult to identify them, and chances of missing them increase. Initially, more importance is given to eliciting the functional requirements where non-functional requirements are discovered at late stages which results in issues related to architectural design (1–3, 5, 6), cost management (9, 22, 23), time management (4, 5, 7, 23), and risk (26) and quality management (5, 7, 27, 28). The problem extrapolates due to manual identification and classification of non-functional requirements (5). The manual classification is quite a time-consuming and laborious process (22, 29). Search function provided by tools is used by typing keywords and searching, which is an exhaustive process and may lead to missing the non-functional requirement (30). The concept of automating or semi-automating this task gained popularity (7, 27). However, this trend of automating the non-functional requirement classification is still young and requires further research for preprocessing, selecting optimum feature sets, acquiring relevant datasets, and using appropriate ML algorithms (8). Moreover, different studies have worked on different categories of non-functional requirements (8) and they considered different types of documents like Certification Commission for Healthcare Information Technology (CCHIT) Ambulatory requirement document (9), Emergency Department Information Systems Functional Document (31), Mercedes-Benz Specification Document (26), and European Union e-Procurement documents (27) from where the non-functional requirements are extracted. Due to different factors, the classification performance reported by different studies is different, and the comparison is not possible because of the element of biasness. Few studies have managed to report the full process of classifying the non-functional requirements from the requirement document (8). Table 1 shows various techniques which are adopted by different studies for the classification of non-functional requirements.

**Table 1:** Non-functional requirements classification review.

**Reference**	**Machine learning algorithm**	**Dataset**	**Limitations**
Shreda and Hanani (30)	Convolutional Neural Network	PURE dataset sample	Only 5 non-functional requirement are considered, Probability of biased result due to 2 fold cross validation
Dias Canedo and Cordeiro Mendes (7)	Logistic Regression	PROMISE_exp	Classification error of 25% on average is reported which needs to be minimized
Younas et al. (32)	K Nearest Neighbor	PROMISE	Precision is found as 50.65%, Recall as 41.11% which indicates quite low classification performance.
Lu and Liang (29)	Bagging	21969 user review sentences	Only four non-functional requirement are considered
Kurtanovic and Maalej (33)	Support Vector Machines	PROMISE	Precision is found to be 78.25% which needs be further improved
Maiti (27)	K Nearest Neighbors	EU Procurement documents, NFRM data set, PROMISE	Datasets used for validation contains only 56 and 78 requirements which are very limited Validation is done only on EU procurement documents
Riaz et al. (31)	K Nearest Neighbors	CCHIT, ED, NU, OSCAR	Validation is done specifically on health care domain documents Only security related requirement are considered
Knauss and Ott (26)	Support Vector Machines	2,000 requirements in Mercedes-Benz specifications	Non-Functional requirement types are not mentioned Validation is done only on automotive industry domain document
Rashwan et al. (23)	Support Vector Machines	Corpus with 3064 annotated sentences	Only 5 non-functional requirement are considered Chances of biased results due to 6 cross fold validation
Slankas and Williams (9)	Support Vector Machines	CCHIT Ambulatory Requirements, iTrust, PROMISE	Performance is low in terms of precision and recall which are only 72.8 and 54.4%, respectively
Casamayor et al. (4)	Expectation Maximization with Naïve Bayes	PROMISE	Only accuracy of 75% is reported
Hussain et al. (22)	Decision Trees	PROMISE	Non-Functional requirement types are not stated
Cleland-Huang et al. (5)	Naïve Bayes	PROMISE, SIEMENS IET Datasets	Classification precision is 12.4% which is very low

### 2.1. Non-Functional Requirements Classification Using Naive Bayes

One of the techniques to classify the non-functional requirements from the requirement document is by Naive Bayes (5). In this technique, the probability is calculated to classify non-functional requirements. Nine non-functional requirements are focused on, and the PROMISE dataset is used. For evaluation, leave one out cross-validation is performed having 15 iterations. The classifier's performance is very poor precision, which is 12.4%. The classification error of the above classifier indicates that it needs improvement.

Similarly, in another study, the Naive Bayes algorithm is used to classify the non-functional requirement but with Expectation-Maximization (4). Non-functional requirements categories in which the data is classified are 9. The classifier's performance in terms of accuracy is reported to be about 75%.

### 2.2. Non-Functional Requirements Classification Using Decision Trees

Decision trees are also used to classify the non-functional requirements (22). PROMISE dataset is used, and 10-fold cross-validation is performed for evaluation. The accuracy of classification is 98.56%. The study above has not mentioned the types of non-functional requirements.

### 2.3. Non-Functional Requirements Classification Using Bagging

Research classifies the non-functional requirements from the requirement document by using machine learning ensemble meta-algorithm Bagging (29). The dataset consisted of 21,969 user reviews. The evaluation is performed using 10-fold cross-validation. The classifier's performance in terms of precision is found to be 71.4% and recall of 72.3%. The non-functional requirements types that are considered by the study are 4. First of all, the types of non-functional requirements considered are only four. Furthermore, the performance of the classifier is relatively low.

### 2.4. Non-Functional Requirements Classification Using SVM

In one research, a SVM is used to extract the non-functional requirements from the requirement document (23). In the technique, the documents are first preprocessed. After preprocessing, SVM is applied. Cross-validation is applied for validation in which the dataset is divided into subsets, and validation is performed in iterations. One subset is used for validation in each iteration, and the remaining subsets are used for training. The performance of the classifier is evaluated using 6-fold cross-validation. The performance on the PROMISE corpus in terms of precision is 77%. The non-functional requirements types that are considered are 5. In terms of performance, there is still work that needs to be done to increase the precision. The Sequential Minimal Optimization (SMO) algorithm is used for classifying the non-functional requirements from the requirement document (9). First, preprocessing is done on text, then SMO is applied. The evaluation is performed using 10-fold cross-validation. The performance is found to be 72.8% in terms of precision. The precision of the classifier needs improvement.

Another study reported using the SVM algorithm to classify the non-functional requirements from the specification of Mercedes Benz (26). This approach is semi-supervised. Manual evaluation is performed. The performance of the classifier in terms of precision is >60%. The research is done using an automotive industry document; there is no surety about how the classifier performs in other industry specifications. SVM is used by one more study to extract the non-functional requirements (33). In this technique, the documents are first preprocessed, then applied SVM. The performance of the classifier is evaluated using 10-fold cross-validation. PROMISE dataset is used for classification. The non-functional requirements which are considered by the study are only 4. The performance in terms of precision is found to be 78.25%.

### 2.5. Non-Functional Requirements Classification Using KNNs

One of the studies focused on classifying security-related requirements (31, 34, 35). In the study, first preprocessing is done, then KNN is applied. The types of security requirements into which the sentences are classified are 7. The evaluation is performed using 10-fold cross-validation. The classifier's performance in terms of precision is found to be 82%. The above study focused on only security-related requirements, not other non-functional requirements.

In literature, another study used KNN to classify the non-functional requirements (27). In the article, first, the text is preprocessed, then KNN is applied. The classifier's performance is found to be 97.73% in terms of precision. The datasets used for evaluation contain requirement documents with 57 and 88 non-functional requirement sentences. The data set used for validation has a minimal number of non-functional requirements.

One of the research used the KNN algorithm concept to classify the non-functional requirements (32). The dataset used for classification is the PROMISE dataset. The classifier's performance is evaluated by iterating 14 times using different threshold values. The classifier's performance in terms of precision is found to be only 50.65%.

### 2.6. Non-Functional Requirements Classification Using LR

Logistic regression is used by research to classify non-functional requirements (7). This algorithm works based on the probability function. The data set used the PROMISE_exp dataset. The types of non-functional requirements considered for classification purposes are 11. The classifier's performance is calculated by performing 10-fold cross-validation, only 75% in terms of precision.

### 2.7. Non-Functional Requirements Classification Using Convolutional Neural Network (CNN)

One study proposed CNN for classifying the non-functional requirements from the requirement document (30). We focus on a total of 5 non-functional requirements. The text is first preprocessed in the study, then CNN is applied. A PURE dataset which consists of 1,247 requirement sentences, is used. The classifier's performance is calculated by performing 2-fold cross-validation, and it is found to be 92.2% in terms of precision.

Similarly, various techniques are proposed to ensure the security of IoT-oriented health care systems. One technique proposed is based on a machine-learning algorithm that uses a biometric framework for ensuring the security of sensitive data (36). In this technique, electrocardiogram (ECG) signals are used to extract the features used by a machine-learning algorithm. The proposed system is based on a multilayer perception model, where in order to secure the signal and protect it from possible interference, a secure communication channel is developed. The authentication process is carried out in the testing phase by considering unique generated EIs from the ECG and coefficients from polynomial approximation.

One study highlights the usage of the biometric cryptosystem (BCS) for ensuring security (37). The proposed approach considers Artificial Neural networks (ANN) to analyze the signal energy variations of implanted devices. The inertial measurement units (IMU) are fixed inside the implanted devices, which help detect signal energy changes. In this technique, ANN is trained so that the first sensor is placed on the chest, and then signals are processed by the algorithm. ANN in the proposed technique comprises three layers: an input layer, an output layer, and a hidden layer comprising 10 hidden nodes. The security is achieved by a 128-bit key generated by gait cycles. This key is difficult for hackers since it cannot be achieved by a modern attacking mechanism like dictionary attacks.

In research study, a novel machine learning-based security framework is proposed to detect malicious activities in healthcare systems (38). The proposed framework named Health Guard is developed by considering four machine learning algorithms: KNN, RF, Decision Trees, and ANN. The malicious activities are detected by observing the vital signals of the implanted devices and then correlating the vitals to identify the variations in the patient body. The classifiers are developed by training using nine databases of eight smart implantable devices and by considering activities, among which 7 are normal user activities, and five are disease-related. The validation process is conducted by testing through only three threats: denial of Service (DoS) attacks, tempered medical devices, and false data injection.

The literature review reflects the limitations of the current study. The performance of some techniques is quite average (1, 6, 26). The non-functional requirements classes considered by some studies are very few (4, 23, 31). The dataset contains limited non-functional requirement sentences for training and testing purposes (2, 3, 9). Studies have mostly considered classifiers, which are developed using single machine learning algorithms (8). Different combinations of hybrid and ensemble classifiers are barely considered.

## 3. Research Methodology

In order to find the relevant features and determine the ML algorithm that achieves better accuracy for the classification of non-functional requirements, the research answers the following research questions:

- **RQ.1**. Which features help to increase the accuracy of non-functional requirement classification?
- **RQ.2**. Which machine learning algorithm classifies non-functional requirements with better accuracy?

The research method used for the validation of the ML algorithm is experimentation (39). Experimentation is a systematic method that involves observation, manipulation, and control to generate accurate and reliable results. It is used to study the cause-effect relationship and prefers standardizing tools for maintaining control and achieving precision. It helps develop automatic techniques and evaluate the accuracy of classifying the non-functional requirements while controlling variables like data used for classification, number of non-functional requirements to be classified, and validation method adopted. Simulation, which is similar to experimentation, is not adopted because of the unavailability of its immense requirements to simulate the technique correctly and because of more chances of uncertainty in results due to its abstract nature (40).

PROMISE_exp dataset is used for classification (7). This dataset contains requirements from real projects, and it is the expanded form of the existing PROMISE dataset. The use of a large dataset also helps in generalizing the results (7). Eleven non-functional requirements are chosen for classification: Availability, Legal and Licensing, Look and Feel, Maintainability, Operability, Performance, Scalability, Security, Usability, Fault Tolerance, and Portability. These non-functional requirements are the common non-functional requirements in many projects and are also misclassified and ignored due to their ambiguous nature (41). The ambiguity level is reduced in this research by using features relevant to non-functional requirements. In addition to this, the PROMISE_exp dataset also supports learning on these types of non-functional requirements (7).

Bag of Words and TF-IDF are used separately to answer the first research question and find the relevant features. The reason for using these methods for finding relevant features is that they work well for classifying the non-functional requirements (7, 8). Moreover, both methods cover different weighing features, and experimenting with both methods helps find more relevant features. BoW ignores the sequence of words; however, in the case of non-functional requirement classification, some algorithms can work well irrespective of the order of the information. BoW method weighs the features according to their number of occurrences in all documents (29) while the TF-IDF method gives more weight to those features which have less overall frequency but more frequency in a specific document (4, 7). Some highly relevant features are also added to help rule-based learning, part of the hybrid KNN-rule based ML algorithm.

**Figure 1:**
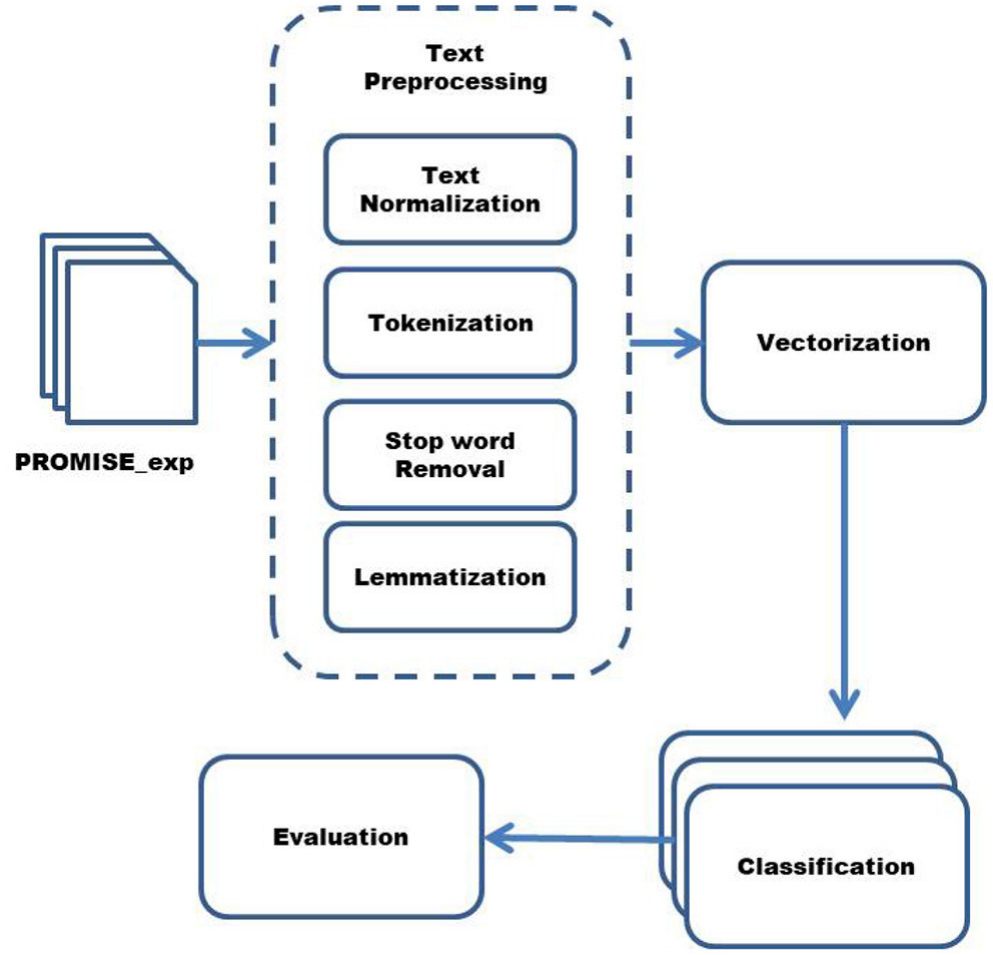
Steps of classifying non-functional requirements.

To answer the second research question and find the ML algorithm that helps classify non-functional requirements with better accuracy, LR, SVM, MNB, KNN, the ensemble made from these algorithms, RF and hybrid KNN-rule based ML algorithms are applied. The reason for choosing these ML algorithms for classifying the non-functional requirements is because these supervised ML algorithms perform better than unsupervised or semi-supervised ML algorithms (8). The algorithms are also selected while keeping in front the dataset, which is used to classify non-functional requirements. To evaluate the accuracy of these classifiers, 10-fold cross-validation is performed. The reason for choosing this method of evaluation is that it helps in producing more accurate and unbiased results (4, 23). Furthermore, many studies adopt 10-fold cross-validation method which helps in comparing the results without bias (4, 5, 7, 23). The steps to the classification of non-functional requirements are given in Figure 1.

**Figure 2:**
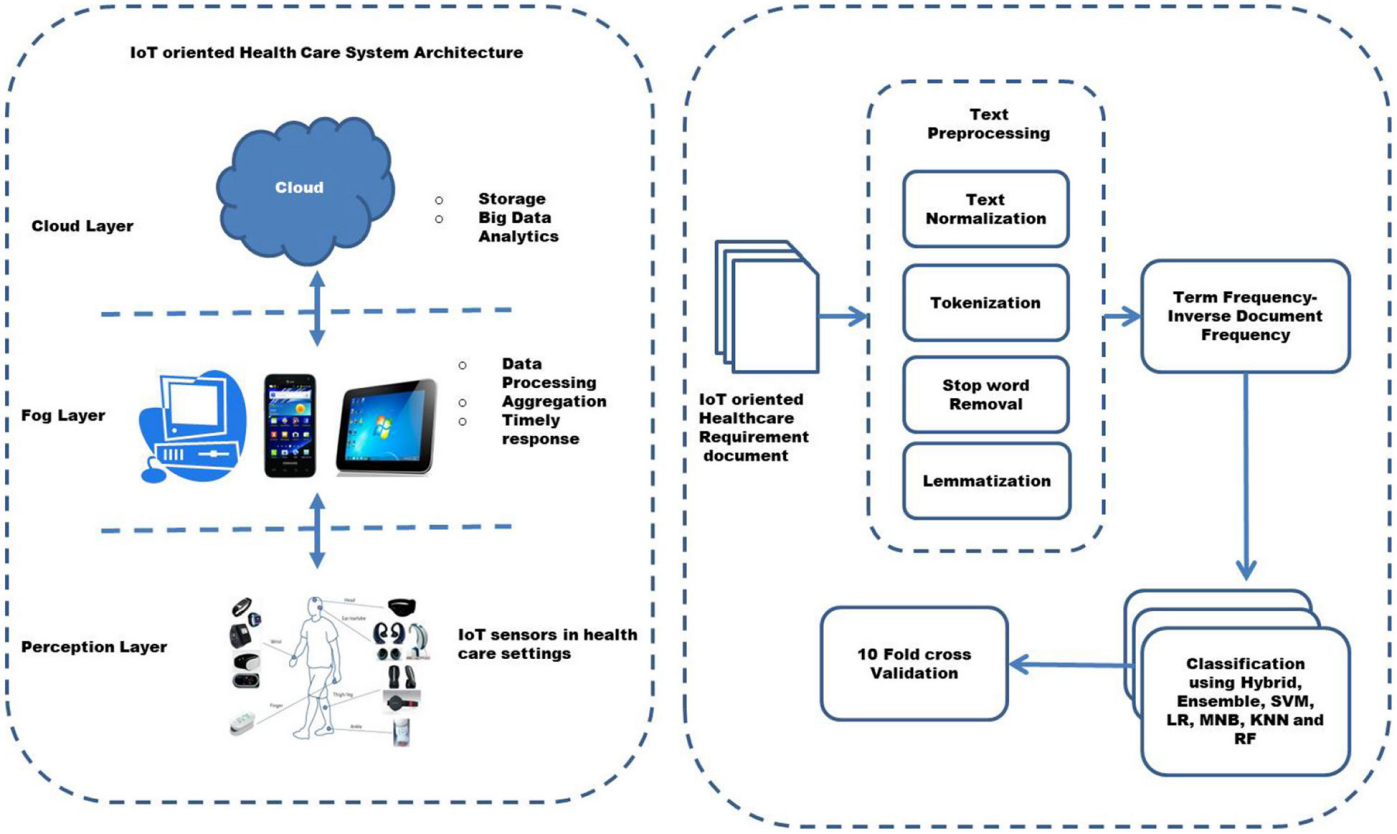
The architectural design of Internet of Things (IoT) oriented Healthcare non-functional requirement classification.

Internet of Things plays a crucial role in the health care systems of the modern era. It provides a facility to monitor, control, and prevent diseases by collecting and processing health-related data through sensors (42). Since these health care systems are critical, it is important to ensure that they work efficiently, encompassing all the required non-functional requirements. Health care systems have compromised performance or security, resulting in the loss of critical health data and even threats and attacks by hackers, which may affect the life of patients (17). An IoT-oriented health care system generally has three layers, which are the perception layer, fog layer, and cloud layer (43–45). The layered architecture is shown in Figure 2. The perception layer includes actuators or devices that help collect sensory data. The fog layer processes the data to produce the required response quickly. The Cloud layer deals with storing the data and big data analytics (44).

### 3.1. Dataset Description

In this research, the PROMISE_exp dataset (46) is used for the classification of non-functional requirements. This dataset is the expanded form of the original PROMISE dataset, which comprised only a total of 625 labeled requirement sentences (21). The expanded form contains more labeled requirement sentences including 444 functional requirements and 525 non-functional requirements (7). The 11 types of non-functional requirements are distributed among the sentences in an unbalanced fashion. Table 2 shows the formulation of this dataset.

**Table 2:** Number of requirements per class.

**Requirement type**	**Number of requirements**
Functional requirement (FR)	444
Availability (A)	31
Security (SE)	125
Usability (US)	85
Look and Feel (LF)	49
Legal and licensing (L)	15
Maintainability (MN)	24
Operability (O)	77
Performance (PE)	67
Scalability (SC)	22
Fault Tolerance (FT)	18
Portability (PO)	12
Total	969

**Figure 3:**
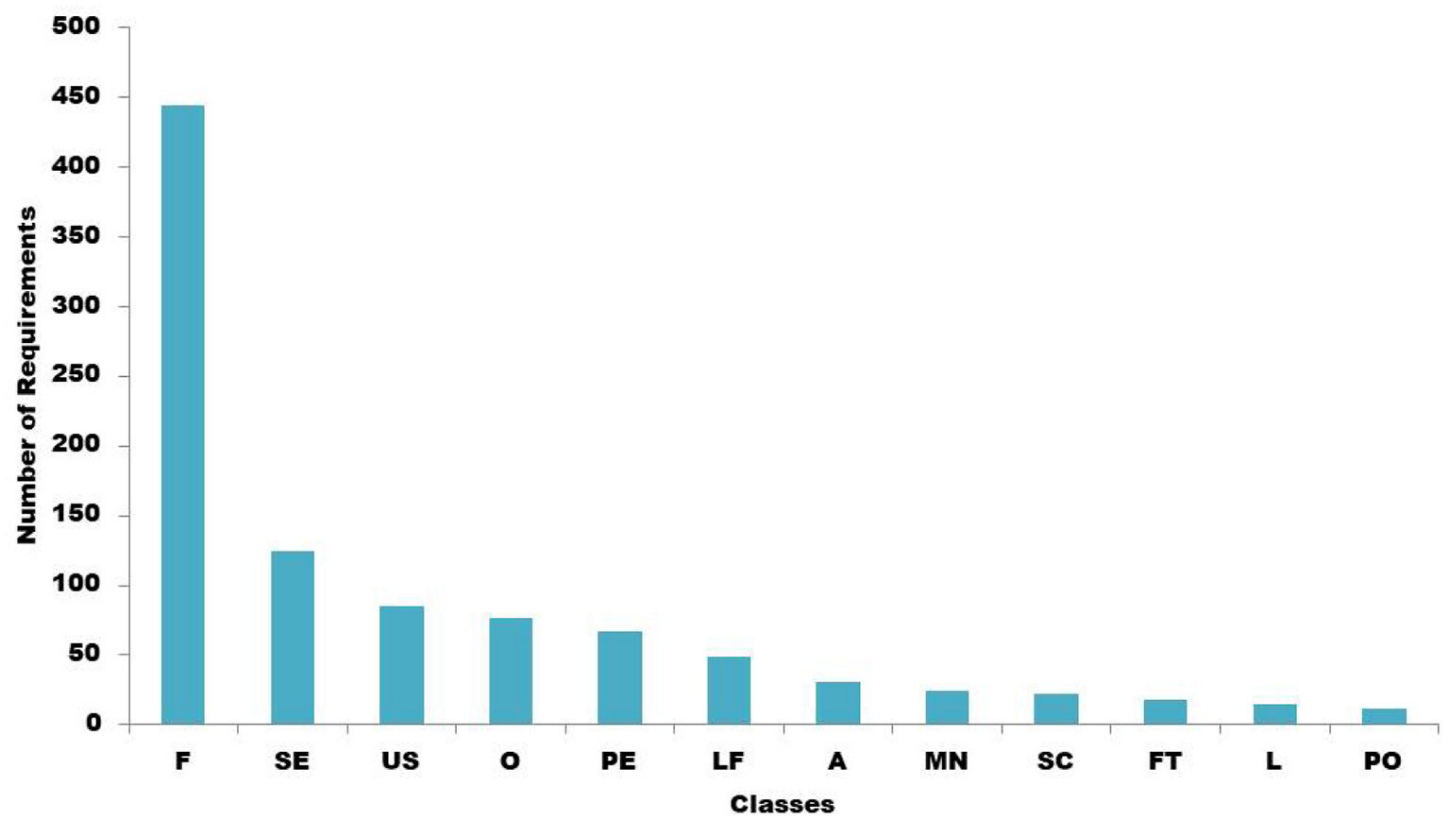
Distribution of requirement classes.

The labeled requirements are distributed in the following manner: availability: 31, security: 125, usability: 85, look and feel: 49, legal and licensing: 15, maintainability: 24, operability: 77, performance: 67, scalability: 22, fault tolerance: 18, portability: 12, and functional requirements which are 444 in number. Figure 3 below shows the distribution of the labeled requirement sentences.

### 3.2. Text Preprocessing

Text preprocessing is the first step in which the data is cleaned to remove redundant and less relevant features (8). In-text preprocessing, first, the data is normalized. During normalization, natural language text is first converted to lower case, then non-alphabetical characters, symbols, and punctuation are removed. Tokenization is performed after normalization, in which text is converted to tokens. Then in the next step, the stop words are removed, which are also less relevant features. Finally, words are lemmatized in which they are converted to their root form in order to remove extra features (7).

### 3.3. Feature Extraction

Feature extraction is the second step in which the preprocessed data is converted into vectors. To extract the features, BoW and TF-IDF are applied. The description of both techniques is given below.

#### 3.3.1. Bag of Words

Bag of Words is a simple technique in which the words are converted into a numerical format based on their number of occurrences. Each feature gets its value equal to the number of times it appears in the requirement sentence. The vector of requirement sentence ‘*j'* is represented in Equation 1.


(1) $$\begin{array}{r} Xj=\left(x_{1,j}\ldots x_{i,j}\ldots xn,j\right) \end{array}$$

In Equation 1, *x*__*i, j*__ represents the weight of the feature which is calculated on the basis of the occurrence of ‘*i'* in the requirement ‘*j*’, whereas ‘*n*’ represents the total number of words (7). When the vectors are created, they are given as an input to the ML algorithms in the next step.

#### 3.3.2. Term Frequency-Inverse Document Frequency

In this technique of vectorization, two metrics play their role. The first metric is the Term Frequency (TF) which represents the number of times a particular word occurs in a requirement. The second metric is the Inverse Document Frequency which is achieved by dividing a total number of requirement sentences by the requirement occurrence number for each word and then applying a logarithmic function on the output (47). Below is the mathematical representation of TF-IDF.


(2) $$\begin{array}{r} TF-IDF\left(term_{i,j}\right)=tf_{i,j}\times idf_{i} \end{array}$$

In Equation 2, *tf*_*i, j*_ shows the frequency of the term *i* in the requirement *j*, where *idf*_*i*_ is the Inverse Document Frequency of ‘*i*’ which is mathematically represented as Equation 3:


(3) $$\begin{array}{r} Idf_{i}=log\frac{total\_requirements}{total\_requirements\_with\_term\_i} \end{array}$$

### 3.4. Machine Learning Algorithms

The machine learning algorithms LR, SVM, MNB, KNN ensemble, RF, and hybrid KNN-rule based ML algorithms are applied to vectorized data. The classifiers are constructed not only by using the monolithic concept but also ensemble and hybrid are also considered since they yield better results according to literature (8).

### 3.5. Evaluation

The performance of the classifiers in terms of accuracy is evaluated using 10-fold cross-validation (7). In 10-fold cross-validation concept, the training set is divided into ten subsets of data which are of almost the exact sizes, and then testing takes place in 10 iterations, wherein each iteration, one fold which comprises 10% of the dataset is left for testing and other nine folds which comprises 90% of the dataset are used for training. In this way, each sample of the data is used once in both training and testing (30). The purpose of performing 10-fold cross-validation is to reduce the chance of biasness in results. In this research, the metric ‘Accuracy’ is considered to get an overall idea of the correct classification made by the classifier. Accuracy of the classifier is the measure of how many correct predictions are made by the classifier in contrast to a total number of predictions as shown in Equation 4.


(4) $$\begin{array}{r} Accuracy=\frac{\left(TP+TN\right)}{P+N} \end{array}$$

Where TP represents the predictions correctly identified as positive, TN represents those correctly identified as negative.

## 4. Experiment

In order to implement the technique and perform the experiment, the Anaconda tool is used. PROMISE_exp dataset is obtained and converted into CSV format to process by the tool. The data is first preprocessed in order to remove useless features. In the first step of data preprocessing, the text is normalized. In the next step, the sentences are converted into tokens. Then in the next step, the stop words are removed. Then in the final step of text preprocessing, words are lemmatized and converted to their root form to remove extra features. After preprocessing, the data is vectorized using BoW and TF-IDF.

For classification of non-functional requirements, 7 ML algorithms are considered, which are LR, SVM, MNB, KNN, ensemble, RF, and hybrid KNN-rule based ML algorithms. The Hyperparameter of the classifiers is tested and set manually by checking their performance. The performance is evaluated in terms of accuracy by using 10-fold cross-validation.

The work in this research differs from the recent study (7) in the sense that this study considers more ML algorithms like RF, Ensemble, and hybrid KNN-rule based ML algorithm. Moreover, the relevant features are also added to increase the performance in terms of accuracy.

The non-functional requirements of an IoT oriented health care system generally include security (17, 42, 44) privacy interoperability, scalability, reliability (17, 44), accuracy, usability (17), performance (17, 44, 48), and maintainability (48). The system must possess these non-functional requirements since missing any critical non-functional requirement results in severe loss of healthcare data and other threats to patients' lives. Thus it is important to extract and classify all the non-functional requirements from the requirement document to be implemented accordingly in the IoT-oriented healthcare system. The manual process of extracting the non-functional requirements is erroneous and laborious. To avoid missing non-functional requirements, this research aims to adopt a machine learning-based approach to automatically classify the non-functional requirements from the IoT-oriented healthcare system's requirement document.

To implement the automatic technique, experimentation is performed. First dataset is created, comprising of functional and non-functional requirements of IoT-oriented health care systems (24, 25). This dataset contains requirements belonging to 8 classes: Accuracy, Reliability, Security and Privacy, Performance, Compatibility, Usability, Functional, and Maintainability. The dataset obtained is then preprocessed. IoT-oriented healthcare systems' requirement sentences are first normalized in the preprocessing stage. The text is converted to lower case in normalization, and punctuation marks and non-alphabetical characters are removed. In the second phase of preprocessing, the text is converted into tokens. In the third phase of preprocessing, stop words are removed. In the fourth phase, lemmatization converts the words into their lemma or dictionary format. After text preprocessing, vectorization is performed using TF-IDF. The features extracted by TF-IDF are then used to create classifiers by applying a machine learning algorithm. The machine learning algorithms adopted in this experiment are SVM, LR, KNN, MNB, RF, Ensemble, and hybrid KNN-rule based machine learning algorithm. For evaluation of classifiers performance, 10-fold cross-validation is performed.

## 5. Results and Discussion

This section presents the experimentation results, which are conducted to find out the relevant features that increase the accuracy of classification of non-functional requirements (RQ1) and explore the ML algorithm, which performs better in terms of accuracy for classification of non-functional requirements (RQ2).

### 5.1. Relevant Features for Non-Functional Requirements Classification

In the experiment stage, two feature extraction techniques are adopted: BoW and TF-IDF. After preprocessing of text and extracting the features with the help of vectorization techniques, the top relevant features which are obtained from both the techniques are shown in Table 3. Furthermore, some coding is also performed to add more features.

**Table 3:** Top relevant features.

**Top relevant features**		
**Number**	**TF-IDF**	**BoW**
1	Must	Must
2	Information	Information
3	Sensitive	User
4	Website	Website
5	Registered	Security
6	Security	Registered
7	Secure	Secure
8	User	Sensitive
9	Third	High
10	Issue	Agreement

In addition to these features obtained by the vectorization techniques, the features which are added according to the relevancy of the type of non-functional requirement are shown in Table 4. These added features helped achieve an accuracy as high as 85% on average, as can be seen, which is achieved by the hybrid KNN rule-based ML algorithm.

**Table 4:** Additional relevant features.

**Requirement type**	**Relevant features**
Availability	availability, available, uptime, mtbf, uptime, system, support, time, hours, whenever, demand.
Legal and Licensing	licensing, legal, law, rule, rules, regulation, law, legal, claim, norm, sarbanesoxley, eula, comply, confirm, meet, violate, violating, violated, follow, agree, requirement, term, condition, accounting, standard.
Fault Tolerance	malfunction, fault, fail, failure, loss, error, warn, alive, handle, prevent, recover, retain, remain, operational, restored, system, back, connection, unavailable, operate, operating.
Security	trusted, unauthorized, malicious, hacker, illegal, secure, security, securely, limited, control, private, password, constrained, constraint, access, accessible, only, need, allow, allowed, require, required, IP address, log, logged, encrypt, encryption, hide, hidden, invisible, password, authentic, PW, attempt, unique, valid, tracking, manner, way, SSL, impose, steal, reveal, infection, virus, threat, trojan, bomb, attack.
Scalability	scale, add, accommodate, include need, increase, release, market, country, country, state, capable, supporting, new, current, customer, user, year, processing, number, grow, multiple, database server, expected, remote.
Look and Feel	look, size, color, shape, texture, position, positioned, located, location, pixel, circle, circular, square, clue, visual, scroll, tool, bar, button, icon, image, buttons, icons, menu, comply, meet, confirm, guideline, standard, display, show, illustrate, simulate, feel, sound, announce, view, grid, graphics, animation, tabbed, schema, attractive, offensive, ethnic, nomenclature, GUI, drilldown, interface.
Usability	show, display, provide, find, locate, icons, tool, tools, toolbar, image, able allow, use, user, customer, scroll, map, web, navigate, navigation, system, friendly, convention, help, ask, section, offer, customize, customization, quickly, easily, quick, easy, understand, entirely, click, select, learn, translate, language, languages, intuitive, feeling, happy, satisfied, content, free, access, accessibility, handicap, disability, one, click, familiar, self-supporting, explanatory.
Performance	perform, check, handle, every, within, second, process, processing balancing, large, simultaneously, parallel, load, response, throughput, lead, cycle, respond, minimum, fast, time, speed, rate, increase, more, high, performance, access, minute, seconds, minutes, space, memory, resource, utilize, utilization, depend, upon, later, returned, asynchronous, asynchronously, overhead, synchronize.
Maintenance	update, maintain, modify, add, integrate, remove, delete, change, replace, upgrade, easily, easy, new, different, release, updated, operate, maintenance, highly, maintainable, configurable, manage, management, function, functionality, component, module, record, data, information.
Operability	operate, exchange, interface, API, APIs, with, without, through, compatible, window, system, os, manage, format, information, data, utilize, run, current, currently, existing, equipment, environment, use, protocol, network, operated, condition, database, store, programming, language, connectivity, evoked, command, platform, prompt, service, stream, navigation, operable.
Portability	portable, portability, support, run, mobile, laptop, tablet, platform, window, os.

**Figure 4:**
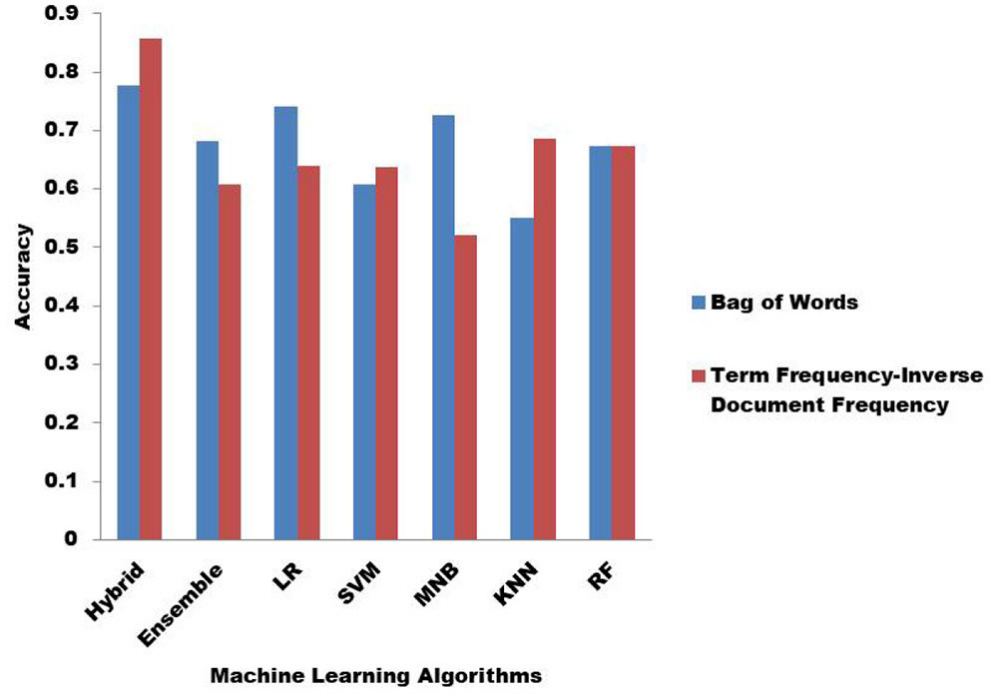
Classification accuracy with Bag of Words (BoW) and Term Frequency-Inverse Document Frequency (TF-IDF).

The performance of the ML algorithms when fed with the features is shown in Figure 4. Figure 4 highlights the effect of BoW and TF-IDF on the classification accuracy of ML algorithms. In the hybrid KNN rule-based ML algorithm application, TF-IDF outperforms BoW to extract more relevant features and provide more accurate results. In the case of the ensemble, BoW provides more relevant features. BoW also outperforms TF-IDF in extracting more relevant features when applied LR and MNB. In the case of SVM and KNN, the features extracted by TF-IDF proved to be more relevant. Both BoW and TF-IDF provide similar accuracy with RF.

### 5.2. Machine Learning Algorithm With Higher Accuracy

**Figure 5:**
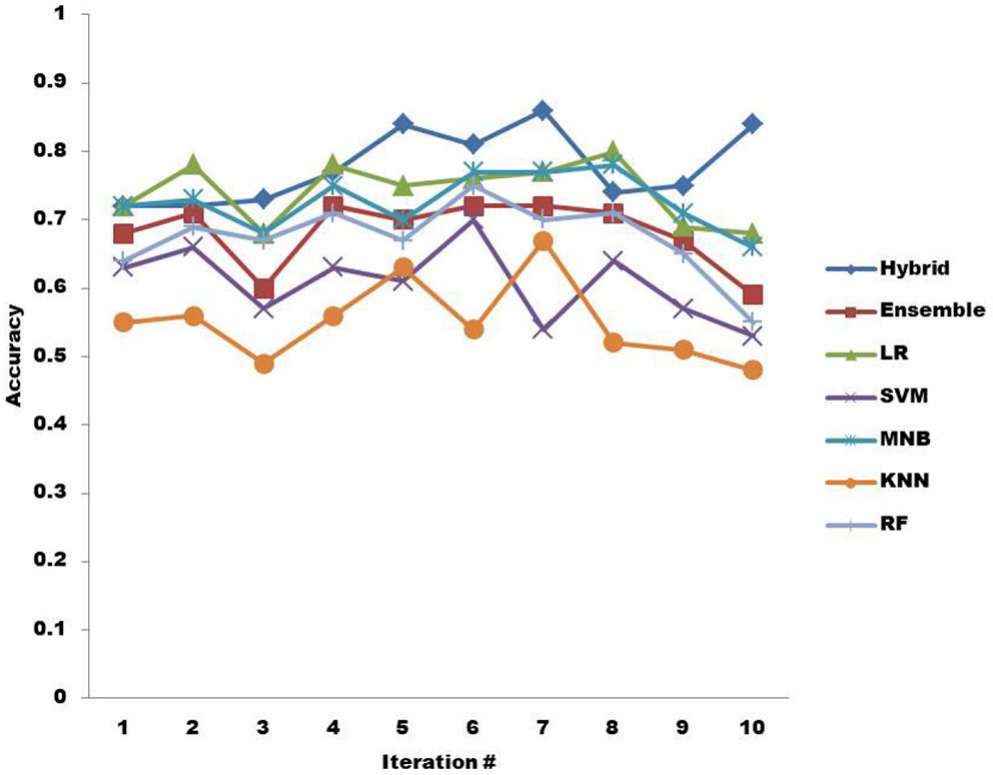
Accuracy of machine learning algorithms with BoW.

The second research question (RQ2) is about finding the ML algorithm that provides more accurate results than others classifying non-functional requirements. In Figure 5, the accuracy provided by all the 7 ML algorithms is illustrated in trend line fashion when BoW is used. There are a total of 10 iterations along which the accuracy is highlighted. The accuracy provided by the hybrid KNN rule-based ML algorithm is the highest overall. The accuracy of the ensemble drops at iteration 3, but overall, it provides almost average classification accuracy compared to the hybrid classifier. On the other hand, LR provides better accuracy than the ensemble overall but is still lower than the hybrid classifier's accuracy. SVM does not show good classification accuracy and performs low overall compared to other ML algorithms except KNN. The classification accuracy provided by MNB is low compared to hybrid and LR overall but still better than other ML algorithms. KNN provides the least good accuracy compared to other ML algorithms overall. RF performs better in terms of accuracy overall than SVM and KNN.

**Figure 6:**
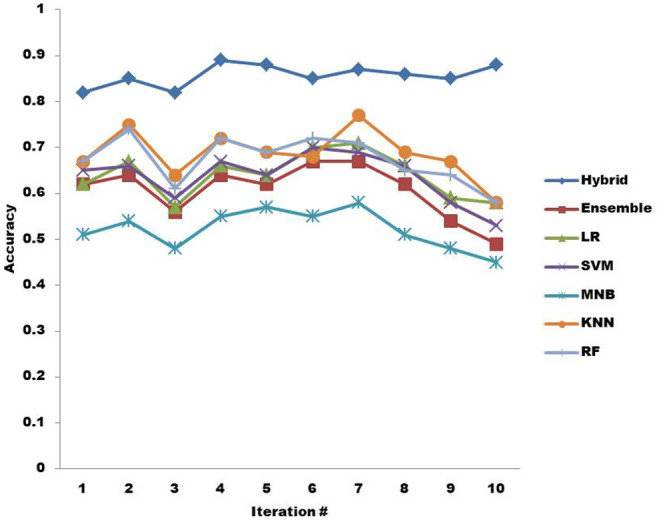
Accuracy of machine learning algorithms with TF-IDF.

In Figure 6, the classification accuracy of all ML algorithms is shown when TF-IDF is adopted. The hybrid KNN rule-based ML algorithm outperforms all other ML algorithms in providing better classification accuracy. Compared to other ML algorithms, ensemble only proved to perform better than MNB in providing better accuracy overall. LR provides better accuracy than MNB, SVM, and ensemble overall, while SVM provides better classification accuracy than just ensemble and MNB. The classification accuracy provided by MNB is low overall compared to all other ML algorithms. KNN, in this case, provides better accuracy than other ML algorithms except for hybrid. RF performs lower in accuracy than hybrid and KNN but provides better classification accuracy overall than other ML algorithms.

The classification results in terms of accuracy are shown in Tables 5, 6. The result in Table 5 clearly shows that hybrid KNN-rule based ML algorithm outperforms other ML algorithms by achieving an average accuracy of 0.778 with BoW. As a result of its nature, a hybrid classifier considers the usage of rules constructed with relevant features, which helps in the classification of non-functional requirements more accurately. Similarly, Table 6 also shows that the highest classification accuracy among all the ML algorithms is achieved by the hybrid KNN-rule based ML algorithm, which is 0.857 accuracy on average. The results show that by using TF-IDF for feature extraction and hybrid KNN-rule based ML algorithm for classification, an average accuracy of 85.7% can be achieved, which is quite promising.

**Table 5:** Classification accuracy of ML algorithms using BoW.

**Classification accuracy in 10 iterations using BoW**											
**Machine learning algorithm**	**1**	**2**	**3**	**4**	**5**	**6**	**7**	**8**	**9**	**10**	**Average classification accuracy**
Hybrid	0.72	0.72	0.73	0.77	0.84	0.81	0.86	0.74	0.75	0.84	0.778
Ensemble	0.68	0.71	0.60	0.72	0.70	0.72	0.72	0.71	0.67	0.59	0.682
LR	0.72	0.78	0.68	0.78	0.75	0.76	0.77	0.80	0.69	0.68	0.741
SVM	0.63	0.66	0.57	0.63	0.61	0.70	0.54	0.64	0.57	0.53	0.608
MNB	0.72	0.73	0.68	0.75	0.70	0.77	0.77	0.78	0.71	0.66	0.727
KNN	0.55	0.56	0.49	0.56	0.63	0.54	0.67	0.52	0.51	0.48	0.551
RF	0.64	0.69	0.67	0.71	0.67	0.75	0.70	0.71	0.65	0.55	0.674

**Table 6:** Classification accuracy of ML algorithms using Term Frequency-Inverse Document Frequency (TF-IDF).

**Classification accuracy in 10 iterations using TF-IDF**											
**Machine learning algorithm**	**1**	**2**	**3**	**4**	**5**	**6**	**7**	**8**	**9**	**10**	**Average classification accuracy**
Hybrid	0.82	0.85	0.82	0.89	0.88	0.85	0.87	0.86	0.85	0.88	0.857
Ensemble	0.62	0.64	0.56	0.64	0.62	0.67	0.67	0.62	0.54	0.49	0.607
LR	0.62	0.67	0.57	0.66	0.64	0.70	0.71	0.66	0.59	0.58	0.640
SVM	0.65	0.66	0.59	0.67	0.64	0.70	0.69	0.66	0.58	0.53	0.637
MNB	0.51	0.54	0.48	0.55	0.57	0.55	0.58	0.51	0.48	0.45	0.522
KNN	0.67	0.75	0.64	0.72	0.69	0.68	0.77	0.69	0.67	0.58	0.686
RF	0.67	0.74	0.61	0.72	0.69	0.72	0.71	0.65	0.64	0.58	0.673

Table 5 shows the average classification accuracy of ML algorithms in 10 iterations when BoW is applied. The hybrid KNN-rule based ML algorithm achieves the highest average classification accuracy, which is 77.8%.

The results of experimentation using IoT-oriented healthcare requirement documents are shown in Table 7. Table 6 shows the average classification accuracy of ML algorithms in 10 iterations when TF-IDF is applied. The hybrid KNN-rule based ML algorithm achieves the highest average classification accuracy, 85.7%.

**Table 7:** Classification accuracy of ML algorithms for IoT oriented healthcare requirements.

**Classification accuracy in 10 iterations using TF-IDF**											
**Machine learning algorithm**	**1**	**2**	**3**	**4**	**5**	**6**	**7**	**8**	**9**	**10**	**Average classification accuracy**
Hybrid	0.73	0.73	0.73	1.00	0.70	0.90	0.60	0.70	0.80	0.70	0.759
Ensemble	0.82	0.64	0.27	0.64	0.80	0.60	0.70	0.70	0.60	0.50	0.627
LR	0.82	0.64	0.27	0.64	0.80	0.60	0.70	0.70	0.60	0.50	0.627
SVM	0.82	0.64	0.27	0.64	0.80	0.60	0.70	0.70	0.60	0.50	0.627
MNB	0.82	0.64	0.27	0.64	0.80	0.60	0.70	0.70	0.60	0.50	0.627
KNN	0.82	0.55	0.45	0.64	0.80	0.70	0.70	0.70	0.50	0.60	0.646
RF	0.82	0.64	0.27	0.64	0.80	0.60	0.70	0.70	0.60	0.50	0.627

The results show that the hybrid KNN-rule based machine learning algorithm outperforms others by showing average classification accuracy of 75.9%. In existing studies, many machine learning-based techniques are adopted to classify the non-functional requirements from the requirement document. However, to our knowledge, this is the first research that solely focuses on IoT-oriented healthcare system requirement documents to classify the non-functional requirements automatically. The automatic technique proposed in this research classifies the non-functional requirements with reasonable accuracy, enabling the development of an excellent IoT-oriented healthcare system.

Certain limitations are present in the approach presented by this research. First, the dataset adopted is minimal and contains only 104 requirements, which may affect the generalizability of the results of this research. Second, the non-functional requirements related to IoT-oriented healthcare systems covered by this research are only 7 in number. Third, the machine learning algorithms considered by this research for classification of non-functional requirements from IoT-oriented healthcare system requirement document are only 7, including only one ensemble and hybrid algorithm and five supervised machine learning algorithms. This research does not cover neural Networks, Semi-supervised or unsupervised machine algorithms.

## 6. Threats to Validity

This section presents the possible threats which affect the validity of this research. The steps taken to mitigate the effect of threats are partially highlighted.

### 6.1. Construct Validity

Construct validity refers to the concept that defines the degree to which the variables measures accurately what they are supposed to measure (41). Many studies consider this measure for measuring the performance of classification, and it is believed to capture the aspect of performance required. In this research, the standard measure “Accuracy” is adopted to measure classifiers' performance.

### 6.2. Internal Validity

Internal validity is related to measuring the extent to which the experimental results are derived from the data and not any unconsidered variables (29). In this research, a threat is considered related to the over-fitting of test data on the machine learning algorithms. The effect of this threat is mitigated by using 10-fold cross-validation.

### 6.3. External Validity

External validity refers to the extent to which the results of this research apply to other settings (29). In this research, the domain and size of the dataset affect the external validity of this research. This threat is mitigated partially by selecting the PROMISE-exp dataset (46), which contains requirement sentences from the software domain, and contains more requirements than the existing PROMISE dataset.

## 7. Conclusion and Future Directions

Non-functional requirements are essential in the RE process since they play an essential role in driving the software architecture and determining the product quality. Since these requirements are written in natural language and often get twined up with the functional requirements, they are often missed or ignored. In order to overcome the difficulties caused due to human manual processes and inadequate tools, this research has focused on using ML algorithms for the automatic classification of non-functional requirements. Since this task of non-functional requirements classification has to be performed accurately, this study has focused on finding relevant features and ML algorithms that can perform this task with great accuracy. In this research, BoW and TF-IDF are used for feature extraction. Further, some features are also added for increasing the accuracy. The ML algorithms adopted in this research are LR, SVM, MNB, KNN, ensemble, RF, and hybrid KNN-rule based ML algorithms. The types of non-functional requirements considered are 11. The dataset used for classification is the PROMISE_exp dataset. In order to compare the accuracy of classifiers created by different ML algorithms and features, experimentation is considered. The experiment results show that by using TF-IDF for extracting the features and hybrid KNN-rule based ML algorithm for classification, an average accuracy of 85.7% can be achieved, which is a pretty excellent performance. It is noteworthy that the accuracy level achieved by this study is higher than the accuracy achieved by a recent study (7) on the PROMISE_exp dataset. Automatic classification of non-functional requirements using high-performing ML algorithms and relevant features helps the Requirement Engineers accurately classify the non-functional requirements. It saves their critical time by using fewer advanced tools and adopting human manual processes.

The research for using the machine learning approach for automatic classification of non-functional requirements with better performance is still in progress. The literature noted that the studies barely consider hybrids and ensembles to classify non-functional requirements. In the future, further study can be done to increase the size of the dataset and incorporate more types of non-functional requirements. Further attention can also be given to creating more custom classifiers by using different combinations of ML algorithms.

## Data Availability Statement

The original contributions presented in the study are included in the article/supplementary material, further inquiries can be directed to the corresponding author/s.

## Author Contributions

IK and SI: conceptualization. SI: data curation. AJ: formal analysis, software, and methodology. IK: funding acquisition. AJ and ZJ: investigation. ZK, AA, and IK: project administration. AA, WB, and IK: resources. ZK and AJ supervision. SI, AA, and WB: validation. WB and ZK: visualization. ZJ and IK: writing—review and editing. All authors contributed to the article and approved the submitted version.

## Conflict of Interest

The authors declare that the research was conducted in the absence of any commercial or financial relationships that could be construed as a potential conflict of interest. The handling editor declared a past co-authorship with one of the authors AJ.

## Publisher's Note

All claims expressed in this article are solely those of the authors and do not necessarily represent those of their affiliated organizations, or those of the publisher, the editors and the reviewers. Any product that may be evaluated in this article, or claim that may be made by its manufacturer, is not guaranteed or endorsed by the publisher.

## References

[1] Iqbal T Elahidoost P Lucio L. A bird's eye view on requirements engineering and machine learning. In: 2018 25th Asia-Pacific Software Engineering Conference (APSEC). Nara: IEEE (2018). p. 11–20.

[2] Vlas R Robinson WN. A rule-based natural language technique for requirements discovery and classification in open-source software development projects. In: 2011 44th Hawaii International Conference on System Sciences. Kauai, HI: IEEE (2011). p. 1–10.

[3] Sabir M Chrysoulas C Banissi E. Multi-label classifier to deal with misclassification in non-functional requirements. In: World Conference on Information Systems and Technologies. Budva: Springer (2020). p. 486–93.

[4] Casamayor A Godoy D Campo M. Identification of non-functional requirements in textual specifications: a semi-supervised learning approach. Inf Softw Technol. (2010) 52:436–45. 10.1016/j.infsof.2009.10.010

[5] Cleland-Huang J Settimi R Zou X Solc P. Automated classification of non-functional requirements. Requirements Eng. (2007) 12:103–20. 10.1007/s00766-007-0045-1

[6] Winkler J Vogelsang A. Automatic classification of requirements based on convolutional neural networks. In: 2016 IEEE 24th International Requirements Engineering Conference Workshops (REW). Beijing: IEEE (2016) p. 39–45.

[7] Dias Canedo E Cordeiro Mendes B. Software requirements classification using machine learning algorithms. Entropy. (2020) 22:1057. 10.3390/e22091057 33286826 PMC7597130

[8] Binkhonain M Zhao L. A review of machine learning algorithms for identification and classification of non-functional requirements. Expert Syst Appl X. (2019) 1:100001. 10.1016/j.eswax.2019.100001

[9] Slankas J Williams L. Automated extraction of non-functional requirements in available documentation. In: 2013 1st International Workshop on Natural Language Analysis in Software Engineering (NaturaLiSE). San Francisco, CA: IEEE (2013). p. 9–16.

[10] Shah A Ahirrao S Pandya S Kotecha K Rathod S. Smart cardiac framework for an early detection of cardiac arrest condition and risk. Front Public Health. (2021) 9:762303. 10.3389/fpubh.2021.762303 34746087 PMC8569303

[11] Javed AR Sarwar MU Beg MO Asim M Baker T Tawfik H. A collaborative healthcare framework for shared healthcare plan with ambient intelligence. Hum Centric Comput Inf Sci. (2020) 10:1–21. 10.1186/s13673-020-00245-7

[12] Awais M Ghayvat H Krishnan Pandarathodiyil A Nabillah Ghani WM Ramanathan A Pandya S. Healthcare professional in the loop (HPIL): classification of standard and oral cancer-causing anomalous regions of oral cavity using textural analysis technique in autofluorescence imaging. Sensors. (2020) 20:5780. 10.3390/s20205780 33053886 PMC7601168

[13] Javed AR Fahad LG Farhan AA Abbas S Srivastava G Parizi RM. Automated cognitive health assessment in smart homes using machine learning. Sustain Cities Soc. (2021) 65:102572. 10.1016/j.scs.2020.102572

[14] Sarwar MU Javed AR. Collaborative health care plan through crowdsource data using ambient application. In: 2019 22nd international multitopic conference (INMIC). Islamabad: IEEE (2019). p. 1–6.

[15] Javed AR Faheem R Asim M Baker T Beg MO. A smartphone sensors-based personalized human activity recognition system for sustainable smart cities. Sustain Cities Soc. (2021) 71:102970. 10.1016/j.scs.2021.102970

[16] Javed AR Sarwar MU Khan HU Al-Otaibi YD Alnumay WS. PP-SPA: privacy preserved smartphone-based personal assistant to improve routine life functioning of cognitive impaired individuals. Neural Process Lett. (2021) 1–18. 10.1007/s11063-020-10414-5

[17] Aruna E Reddy A Sunitha K. Elicitation and analysis of security requirements and patterns for IoT based health monitor. In: Advances in Cybernetics, Cognition, and Machine Learning for Communication Technologies. Singapore: Springer (2020). p. 49–56.

[18] Shabbir M Shabbir A Iwendi C Javed AR Rizwan M Herencsar N. Enhancing security of health information using modular encryption standard in mobile cloud computing. IEEE Access. (2021) 9:8820–34. 10.1109/ACCESS.2021.3049564

[19] Mubashar A Asghar K Javed AR Rizwan M Srivastava G Gadekallu TR. Storage and proximity management for centralized personal health records using an IPFS-based optimization algorithm. J Circ Syst Comput. (2021) 31:2250010. 10.1142/S0218126622500104

[20] Nasir M Javed AR Tariq MA Asim M Baker T. Feature engineering and deep learning-based intrusion detection framework for securing edge IoT. J Supercomput. (2022) 1–15. 10.1007/s11227-021-04250-0

[21] Tóth L Vidács L. Study of various classifiers for identification and classification of non-functional requirements. In: International Conference on Computational Science and Its Applications. Melbourne, VIC: Springer (2018). p. 492–503.

[22] Hussain I Kosseim L Ormandjieva O. Using linguistic knowledge to classify non-functional requirements in SRS documents. In: International Conference on Application of Natural Language to Information Systems. London: Springer (2008). p. 287–98.

[23] Rashwan A Ormandjieva O Witte R. Ontology-based classification of non-functional requirements in software specifications: a new corpus and svm-based classifier. In: 2013 IEEE 37th Annual Computer Software and Applications Conference. Kyoto: IEEE (2013). p. 381–6.

[24] Caballero K. Schedule A: Functional Non- Functional Requirements. betterliving (2017). Available online at: http://mybetterliving.ca/wp-content/uploads/2017/01/Schedule-A-Integrated-IM-IT-Solution-for-Community-Hospice-Services-Functional-Non-Functional-Requirements-100117.pdf.

[25] Sigdel S Yadav R. Software Requirements Specification of E-Health Architecture for Nepal (2019) 11–4. 10.13140/RG.2.2.17207.57764

[26] Knauss E Ott D. (Semi-) automatic categorization of natural language requirements. In: International Working Conference on Requirements Engineering: Foundation for Software Quality. Essen: Springer (2014). p. 39–54.

[27] Maiti R. Capturing. Eliciting, and prioritizing (CEP) non-functional requirements metadata during the early stages of agile software development. In: SoutheastCon 2015. Fort Lauderdale: IEEE (2016).

[28] Yenduri G Gadekallu TR. Firefly based maintainability prediction for enhancing quality of software. Int J Uncertainty Fuzziness Knowl Based Syst. (2021) 29:211–35. 10.1142/S0218488521400122

[29] Lu M Liang P. Automatic classification of non-functional requirements from augmented app user reviews. In: Proceedings of the 21st International Conference on Evaluation and Assessment in Software Engineering. Karlskrona (2017). p. 344–53.

[30] Shreda QA Hanani AA. Identifying non-functional requirements from unconstrained documents using natural language processing and machine learning approaches. IEEE Access. (2021) 1. 10.1109/ACCESS.2021.3052921

[31] Riaz M King J Slankas J Williams L. Hidden in plain sight: automatically identifying security requirements from natural language artifacts. In: 2014 IEEE 22nd International Requirements Engineering Conference (RE). Karlskrona: IEEE (2014). p. 183–92.

[32] Younas M Wakil K Jawawi D Shah MA Mustafa A. An automated approach for identification of non-functional requirements using word2vec model. Int J Adv Comput Sci Appl. (2019) 10:539–47. 10.14569/IJACSA.2019.0100871

[33] Kurtanovic Z Maalej W. Automatically Classifying Functional and Non-functional Requirements Using Supervised Machine Learning. In: 2017 IEEE 25th International Requirements Engineering Conference (RE). Lisbon: IEEE (2017). p. 490–5.

[34] Iwendi C Jalil Z Javed AR Reddy T Kaluri R Srivastava G. Keysplitwatermark: zero watermarking algorithm for software protection against cyber-attacks. IEEE Access. (2020) 8:72650–60. 10.1109/ACCESS.2020.2988160

[35] Srivastava G Deepa N Prabadevi B Reddy MPK. An ensemble model for intrusion detection in the internet of softwarized things. In: Adjunct Proceedings of the 2021 International Conference on distributed Computing and Networking. Nara (2021). p. 25–30.

[36] Pirbhulal S Pombo N Felizardo V Garcia N Sodhro A Mukhopadhyay SC. Towards machine learning enabled security framework for iot-based healthcare. In: 2019 13th International Conference on Sensing Technology (ICST). Sydney, NSW: IEEE (2019). p. 1–6.

[37] Bharadwaj HK Agarwal A Chamola V Lakkaniga NR Hassija V Guizani M. A review on the role of machine learning in enabling IoT based healthcare applications. IEEE Access. (2021) 9:38859–90. 10.1109/ACCESS.2021.3059858

[38] Newaz AI Sikder AK Rahman MA Uluagac AS. Healthguard: a machine learning-based security framework for smart healthcare systems. In: 2019 Sixth International Conference on Social Networks Analysis, Management and Security (SNAMS). Granada: IEEE (2019). p. 389–96.

[39] Goundar S. Research Methodology and Research Method. Wellington: Victoria University of Wellington (2013).

[40] Deist T Patti A Wang Z Krane D Sorenson T Craft D. Simulation-assisted machine learning. Bioinformatics. (2019) 35:4072–80. 10.1093/bioinformatics/btz199 30903692 PMC6792064

[41] Mahmoud A Williams G. Detecting, classifying, and tracing non-functional software requirements. Requirements Eng. (2016) 21:357–81. 10.1007/s00766-016-0252-8

[42] Gelogo YE Hwang HJ Kim HK. Internet of things (IoT) framework for u-healthcare system. Int J Smart Home. (2015) 9:323–30. 10.14257/ijsh.2015.9.11.31

[43] Mohiyuddin A Javed AR Chakraborty C Rizwan M Shabbir M Nebhen J. Secure cloud storage for medical IoT data using adaptive neuro-fuzzy inference system. Int J Fuzzy Syst. (2021) 1–13. 10.1007/s40815-021-01104-y

[44] Calvillo-Arbizu J Román-Martínez I Reina-Tosina J. Internet of things in health: requirements, issues, and gaps. Comput Methods Progr Biomed. (2021) 208:106231. 10.1016/j.cmpb.2021.106231 34186337

[45] Ikram AA Javed AR Rizwan M Abid R Crichigno J Srivastava G. Mobile cloud computing framework for securing data. In: 2021 44th International Conference on Telecommunications and Signal Processing (TSP). Brno: IEEE (2021). p. 309–15.

[46] Lima M Valle V Costa E Lira F Gadelha BF. Software engineering repositories: expanding the promise database. In: Proceedings of the XXXIII Brazilian Symposium on Software Engineering. Salvador (2019).

[47] Amasaki S Leelaprute P. The Effects of Vectorization Methods on Non-Functional Requirements Classification. In: 2018 44th Euromicro Conference on Software Engineering and Advanced Applications (SEAA). Prague (2018). p. 175–82.

[48] Nasiri S Sadoughi F Tadayon MH Dehnad A. Security requirements of internet of things-based healthcare system: a survey study. Acta Inf Med. (2019) 27:253. 10.5455/aim.2019.27.253-258 32055092 PMC7004290

